# Protein Folding: Adding a Nucleus to Guide Helix Docking Reduces Landscape Roughness

**DOI:** 10.1016/j.jmb.2012.08.003

**Published:** 2012-10-26

**Authors:** Beth G. Wensley, Lee Gyan Kwa, Sarah L. Shammas, Joseph M. Rogers, Jane Clarke

**Affiliations:** Department of Chemistry, University of Cambridge, Lensfield Road, Cambridge CB2 1EW, UK

**Keywords:** protein folding, Φ-value analysis, energy landscape, helix bundle, minimal frustration

## Abstract

The elongated three-helix‐bundle spectrin domains R16 and R17 fold and unfold unusually slowly over a rough energy landscape, in contrast to the homologue R15, which folds fast over a much smoother, more typical landscape. R15 folds via a nucleation–condensation mechanism that guides the docking of the A and C-helices. However, in R16 and R17, the secondary structure forms first and the two helices must then dock in the correct register. Here, we use variants of R16 and R17 to demonstrate that substitution of just five key residues is sufficient to alter the folding mechanism and reduce the landscape roughness. We suggest that, by providing access to an alternative, faster, folding route over their landscape, R16 and R17 can circumvent their slow, frustrated wild-type folding mechanism.

## Graphical Abstract

**Figure:**
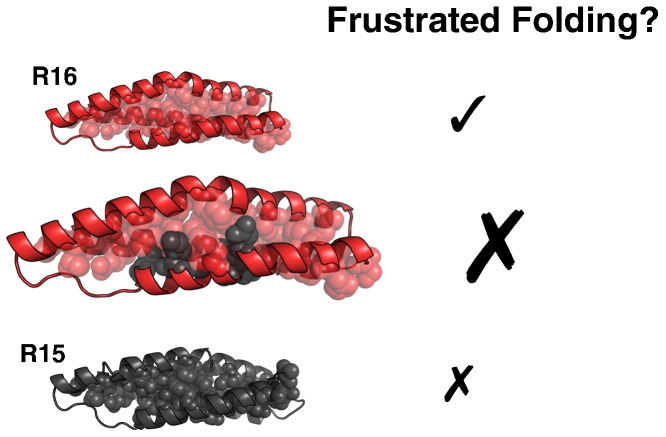

**Research Highlights:**

► Homologous spectrin domains have very different folding behavior. ► Spectrin domain R16 folds slowly over an atypically rough energy landscape. ► We have substituted just five residues from R15 to R16. ► The mutated protein folds via a different folding mechanism (more like R15). ► This results in a faster folding across a smoother (more R15 like) landscape.

## Introduction

**Fig. 1:**
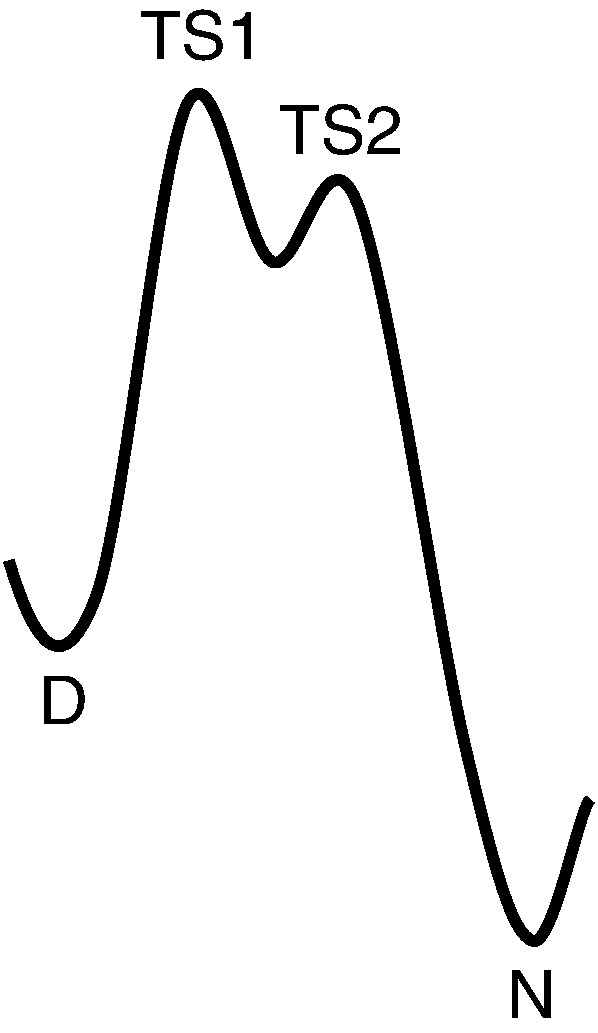
R16 and R17 fold on an energy landscape with two sequential transition states (TSs) and a high‐energy intermediate. At low concentrations of denaturant, the rate‐limiting transition state is TS1.^13^ At TS1, helices A and C dock, establishing the correct topology and register of the long spectrin helices. At TS2, structure condenses and the B-helix starts to pack.^4,5^ It is the folding and unfolding over TS1 that is investigated in this study. The landscape at TS1 is rough and this roughness slows folding by about 5-fold.^6,14^ At TS2, there is no evidence for roughness in the energy landscape (B. G. Wensley and J. Clarke, unpublished data).

Comparative folding studies combined with energy landscape theory have been applied successfully to the 15th, 16th and 17th repeats of chicken brain α-spectrin (R15, R16 and R17).^1–7^ These domains are elongated three-helix bundles with a 106‐residue repeat length.^8–11^ All three have similar structures, stabilities and Tanford β-values, but R16 and R17 fold and unfold some 3 orders of magnitude more slowly than R15.^3,12^ The folding landscapes of spectrin domains are complex. We have previously shown that R16 and R17 are best described as folding on a landscape with a high-energy intermediate and that there are two consecutive transitions states, one early (TS1, rate limiting at low denaturant concentrations) and one late (TS2) (Fig. 1).^3–5,13^ These slow-folding domains have been shown to have a rough energy landscape at TS1, which is responsible in part for the reduction in both folding and unfolding rate constants.^6^ In fact, landscape roughness acts to reduce the folding and unfolding rate constants around 5-fold.^14^ Recent characterization of a single point mutant (E18F) with a landscape that remains as rough as that of its R16 parent protein and folds via the same mechanism but with vastly increased rate constants has identified the remainder (majority) of the “slowing” to traditional effects of burial of charge on the transition state.^14^ For both R16 and R17, although TS1 is rough, the landscape at TS2 is smooth (B. G. Wensley and J. Clarke, unpublished data). R15 probably also has a complex folding landscape, but due to the speed with which it folds, only events in the early part of this landscape can be probed. In contrast to R16 and R17, the early, rate determining TS (equivalent to TS1 in R16 and R17) over which R15 folds and unfolds appears to lack roughness.^7^

Energy landscape theory (introduced in the late 1980s) proposes that evolution has resulted in energy landscapes that are smooth, or unfrustrated (the principle of minimal frustration).^2^ In particular, nonnative interactions are disfavored so that folding can proceed rapidly on a funnel-shaped free energy landscape. However, theory predicts that some frustration, or energetic roughness, may still exist in natural proteins, manifested as small local energetic traps, which will slow folding.^2,15^ These small local kinetic traps are not traditional folding intermediates that accumulate but are manifested as “internal friction”. To date, landscape roughness effects on folding dynamics have not been seen for any other domains of comparable size and folding timescale, although roughness has been predicted in theoretical studies.^2,15–21^

The similarities between R15, R16 and R17 suggested a sequence-specific origin for this atypical roughness. The behavior of two fast-folding core-swapped spectrin chimeric domains, R16o15c (outside of R16 with the core of R15) and R17o15c, shows this to be the case as both have ~ 80% sequence identity with their slow-folding parental domain but have reduced transition state roughness^6^ and an altered folding mechanism: from a framework, diffusion–collision mechanism in the slow‐folding wild-type parent domains to a more nucleation–condensation-like mechanism as is seen in R15.^22^

Here, we take the core-swapping strategy further, in order to probe the mechanistic basis for the landscape roughness observed in R16 and R17 folding. We investigate eight more core-swapped domains and identify a subset of just five residues on the A‐helix that are sufficient to induce a decrease in landscape roughness. We use Φ-value analysis to probe the folding mechanism of one of these fast‐folding versions of R16 and show that the roughness can be diminished by providing an alternative folding pathway via a stable folding nucleus.

## Results

### The minimal fast‐folding core

**Fig. 2:**
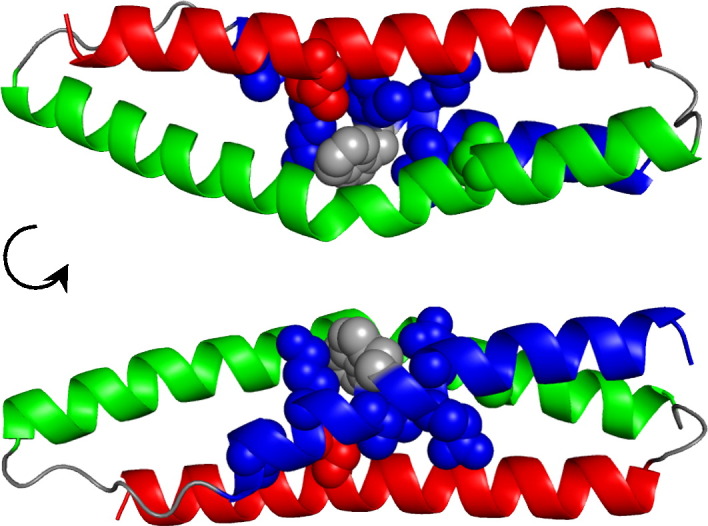
The minimal core residues R16 (taken from 1u4q^23^) with the seven residues initially defined as the minimal core shown as space‐filling models. The A‐helix is blue (and has five of the minimal core residues), the B‐helix is green (and has one) and the C‐helix is red (and also has one). Trp21, which is at the center of this cluster, is shown in gray.

**Fig. 3:**
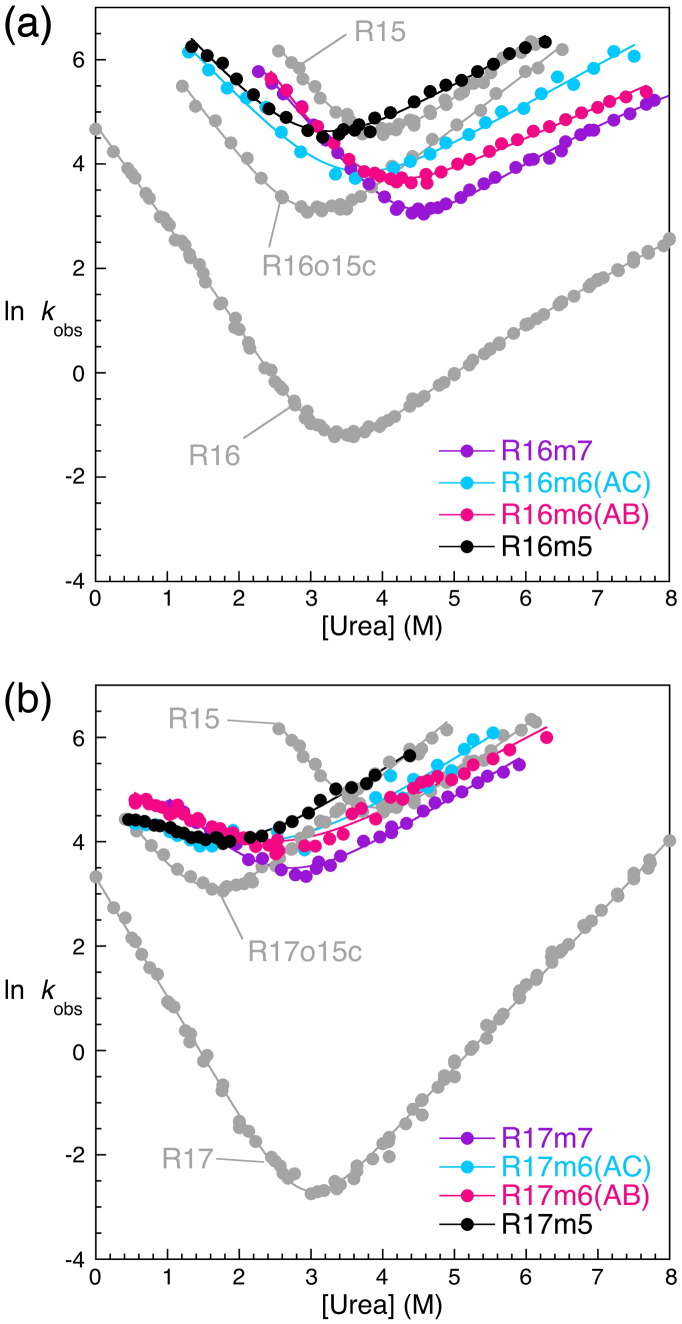
Kinetics of the R16 and R17 minimal core variants. (a) Chevron plots for the R16 minimal core variants show that these fold significantly faster than R16 and at a rate similar to that of R16o15c. (b) Chevron plots for the R17 minimal core variants show that these also fold significantly faster than R17 and at a rate similar to that of R17o15c, although they are significantly less stable than R17 and show reduced values of *m*_*k*f_.

Previously, we inserted the entire core of R15 into R16 and R17 to produce faster‐folding core-swapped domains with reduced landscape roughness. We noticed that there are 10 core residues that are identical in R16 and R17 but are different in R15 and used these to narrow down the residues responsible for this reduced roughness. Of these, seven are in close proximity to one another (Fig. 2). In R16, these seven residues were mutated to the side chain found at this position in R15 to produce the minimal core-swapped domain R16m7 (m, a minimal core-swapped variant of the named parental domain; 7, seven residues are different to the parent domain). These mutations comprise five in helix A (E18F, E19D, I22L, K25V and V29L), one in helix B (V65L) and one in helix C (L97I). R16m7 was fully folded, as judged by size-exclusion chromatography, circular dichroism spectroscopy and cooperative unfolding in urea. It has a comparable thermodynamic stability to R16, despite showing a slight reduction in *m*_D–N_ (Table 1 and Table S1). The folding kinetics of R16m7 are shown in Fig. 3a, Table 1 and Table S1. The effect of thermodynamic stability on both folding and unfolding rate constants can complicate the interpretation of how fast a domain folds. To correct for differing stabilities, we have used the rate constant at an equilibrium free energy, ∆*G*_D–N_, of 0.0 kcal mol^−^ ^1^ (*k*^∆*G* = 0^) to judge the folding kinetics of these spectrin domains. (*k*^∆*G* = 0^ also avoids the need for long extrapolations to reach rate constants at zero M denaturant.) For R16m7, *k*^∆*G* = 0^ = 14 (± 4) s^−^ ^1^, about 2 orders of magnitude faster than the R16 value [*k*^∆*G* = 0^ = 0.19 (± 0.01) s^−^ ^1^] despite being only seven residues different (Table 1).

Two strategies were adopted to dissect the effect of individual residues. First, of the seven residues mutated in R16m7, we investigated the two conservative substitutions that are not in helix A, V65L (B-helix) and L97I (C-helix) (Fig. 2). These two residues were returned to those seen in R16 either alone or in concert. Residue 97 was returned to Leu to produce a protein with mutations only in helices A and B [R16m6(AB)], residue 65 was returned to Val to produce R16m6(AC) and both were returned to create R16m5, where only the five residues on the A‐helix are different to R16. These variants all behave in a way similar to that of R16m7; thus, R16m6(AC), R16m6(AB) and R16m5 all still fold and unfold considerably faster than R16 (Fig. 3a, Table 1 and Table S1). The return of V65 and L97 either alone or in concert did not significantly slow the folding of these minimal core-swapped R16 variants. Second, we changed each of the residues in the A-helix, individually, in the background of the wild-type protein.^14^ Mutation of three of the residues has little effect on the folding kinetics (E19D, I22L and V29L), whereas mutation of two (E18F and K25V) speeds folding; in the case of E18F, this speeding of folding and unfolding is significant. However, and most importantly, this speeding of folding is not accompanied by either a reduction in landscape roughness or a change in pattern of Φ-values (and thus folding mechanism).^14^

Since the residues exchanged in R16m7 are identical in R16 and R17, the equivalent R17 minimal core-swapped domains [i.e., R17m7, R17m6(AB), R17m6(AC) and R17m5] were produced (Fig. 3b, Table 1 and Table S1). These R17 minimal core swaps are considerably destabilized, with reduced *m*_D–N_ values and folding *m*-values, *m*_*k*f_, relative to R17. These results are consistent with the behavior observed for the previously studied full core-swapped domain R17o15c. The *k*^∆*G* = 0^ for all these minimal core swaps is increased by ~ 3 orders of magnitude compared to the parental R17 (Table 1). The R16 minimal core variants are more attractive for further study because they are considerably more stable than those of R17.

The aim of the study reported here was to investigate the link between a rough energy landscape and the mechanism for folding. We investigate roughness using solvent viscosity dependence and folding mechanism using a comparative Φ-value analysis. We have previously shown that comparisons of the Φ-values of the C-helix are the clearest indicator for different folding mechanisms in spectrin domains^4,6,7^; thus, we wished to use a protein with an entirely wild type C-helix for our comparative study. Since our results showed that the conservative mutation L97I in the C-helix does not slow the folding of the minimal core‐swapped domain, we chose to use the core‐swapped domain with the changes in the A‐helix and in the B‐helix only, R16m6(AB) for further study. This is the most stable of the minimal core-swapped variants.

### The effect of solvent viscosity on the folding of R16m6(AB)

The effect of solvent viscosity on the folding of R16m6(AB) was determined as a measure of folding landscape roughness. The approach used is based on that previously applied to R15, R16, R17, R16o15c and R17o15c, which uses an empirically derived formulation of Kramers' theory^24^ describing folding as a diffusive process over an energy surface, where the folding or unfolding rate constant (*k*) is dependent on η (the solvent viscosity), σ (the internal friction of the protein), ∆*G*^TS^ (the height of the energy barrier) and *C* (a temperature‐ and solvent‐independent term, comprising all components of the pre-exponential factor except the friction terms)^6,24–26^:

(1) $$k=\frac{C}{\eta +\sigma }\text{exp}\left(\frac{-\Delta G^{\text{TS}}}{RT}\right)$$

**Fig. 4:**
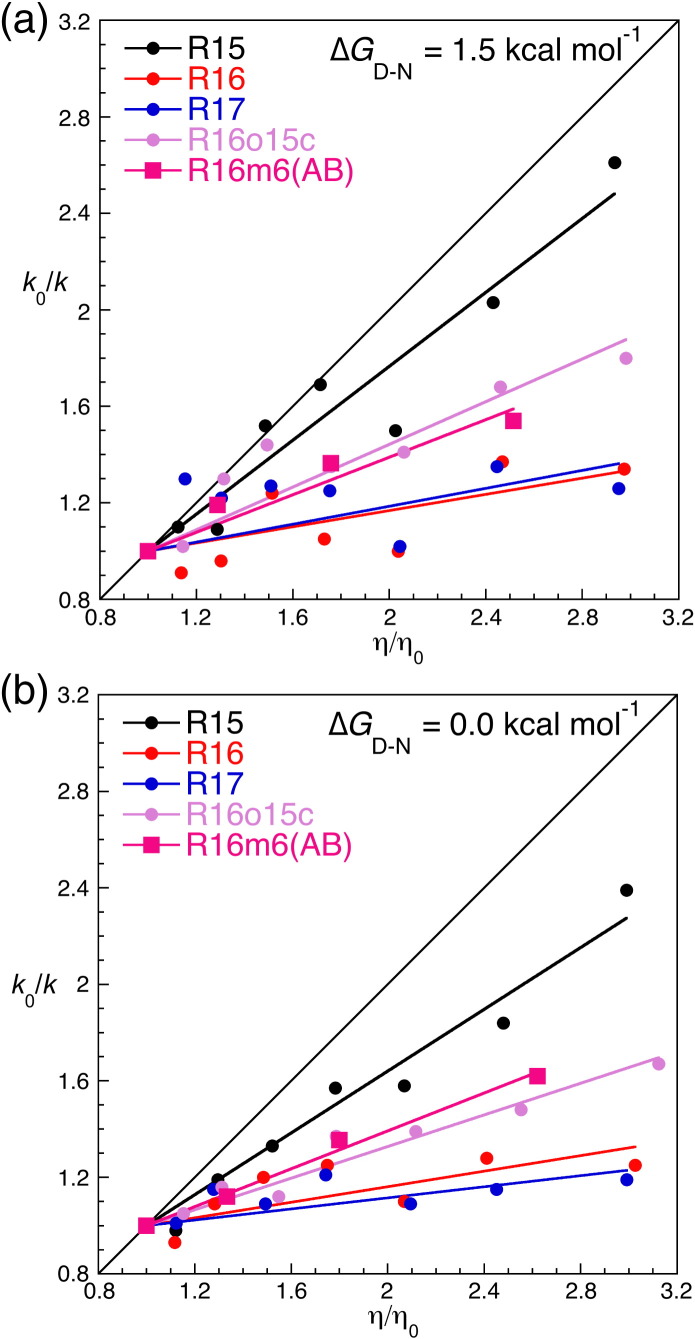
The folding of R16m6(AB) has a greater dependence on solvent viscosity than does R16. Dependence of the relative folding and unfolding rate constants (*k*_0_/*k*) on relative solvent viscosity (η/η_0_). Values of *k*_f_ determined at (a) ∆*G*_D–N_ = 1.5 kcal mol^−^ ^1^ and at (b) ∆*G*_D–N_ = 0.0 kcal mol^−^ ^1^, where *k*_f_ = *k*_u_. The folding and unfolding of R16m6(AB), like R16o15c, has an intermediate dependence on solvent viscosity.

This formulation assumes that solvent friction and internal friction are additive. At a constant ∆*G*^TS^ when internal friction is negligible (i.e., σ ≪ η), *k* is inversely proportional to solvent viscosity and the slope of the plot of relative solvent viscosity *versus* relative rate constant should be close to unity. This should be the case for reactions with a smooth energy landscape and was seen for R15 (and other similar small proteins^17–22^). In contrast, for R16 and R17, *k* shows little dependence on solvent viscosity. They have substantial values of internal friction (σ) significantly larger than solvent viscosity (η); thus, the effect of altering the solvent viscosity on the pre-exponential factor is small. R16o15c shows an intermediate dependence, and R17o15c shows a strong R15-like solvent viscosity dependence (Fig. 4 and Table S2). Small‐molecule viscogens such as glucose tend to stabilize proteins and thus alter ∆*G*_D–N_ and ∆*G*^TS^. To offset this, we apply the widely used isostability approach.^17–22,28,29^ The stabilizing effect of the glucose is counteracted using a chemical denaturant, and the stronger denaturant guanidinium chloride (GdmCl) is generally required, rather than urea. *k* is determined when ∆*G*_D–N_ = 0.0 and 1.5 kcal mol^−^ ^1^, for each glucose concentration. A value for the magnitude of σ can also be determined by rearranging Eq. (1) (see Materials and Methods). Although this approach has been criticized,^30^ the strength of our comparative studies is that any caveats apply equally to the three parent proteins and to their core-swapped derivatives. (For a full discussion of this approach in determining the magnitude of internal friction, see Supplementary Information for Wensley *et al.*^6^ and Borgia *et al.*^15^).

Folding and unfolding kinetics as a function of GdmCl and glucose concentrations are shown in Fig. S1a (see Materials and Methods for fitting details). The effect of solvent viscosity on the folding of R16m6(AB) is shown as relative solvent viscosity *versus* relative rate constant plots (Fig. 4 and Table S2). The average slope of the two viscosity plots for R16m6(AB) is 0.39 (± 0.01), compared with 0.20 (± 0.07) for R16, 0.75 (± 0.10) for R15 and 0.38 (± 0.06) for R16o15c. The average value of σ for R16m6(AB) is 1.6 (± 0.2) cP (Fig. S1b and Table S2), again, similar to that of R16o15c [2.1 (± 0.2) cP]. The internal friction seen for both R16o15c and R16m6(AB) is considerably lower than that for R16 [3.9 (± 0.8) cP] but is greater than that for R15 [0.26 (± 0.09)]. The six residues mutated in R16m6(AB) have reduced the landscape roughness of R16 to levels comparable with the full core-swapped R16o15c, despite the 95% sequence identity between R16 and R16m6(AB).

### The transition‐state structure of R16m6(AB)

When the Φ-values of R15 are compared with those of R16 and R17, there are clear differences in the pattern of Φ-values in the A‐helix and in the C‐helix. In particular, the pattern of Φ-values in the C-helix suggests very different folding mechanisms (Fig. S2). Thus, to determine whether the reduced roughness in the minimal core-swapped domain is related to a shift in folding mechanism, we performed a Φ-value analysis of the C‐helix. Identical conditions and mutations were used to those in the Φ-value analysis of R16.^4^ Two types of substitution were made: (i) core mutations using a nondisruptive deletion mutation to probe the formation of tertiary structure at the transition state and (ii) surface Ala–Gly helix scanning mutations to probe helix formation.^31,32^

Equilibrium denaturation curves were used to determine [urea]_50%_, *m*_D–N_ value and *ΔG*_D − N_^H_2_O^ of all mutants (Table S3). Although we have not determined the structure of R16m6(AB), the ∆∆*G*_D–N_ for most of these mutants is similar to that for the same mutation made in R16. This suggests only small structural changes as a result of the mutations. The exceptions to this are F90A, W94F and L97A, which are less destabilizing in R16m6(AB) than in R16 (Fig. S3). These three are at the center of the C‐helix and pack against the mutations made in the A‐helix to create R16m6(AB) where we might expect the packing to be significantly different.

**Fig. 5:**
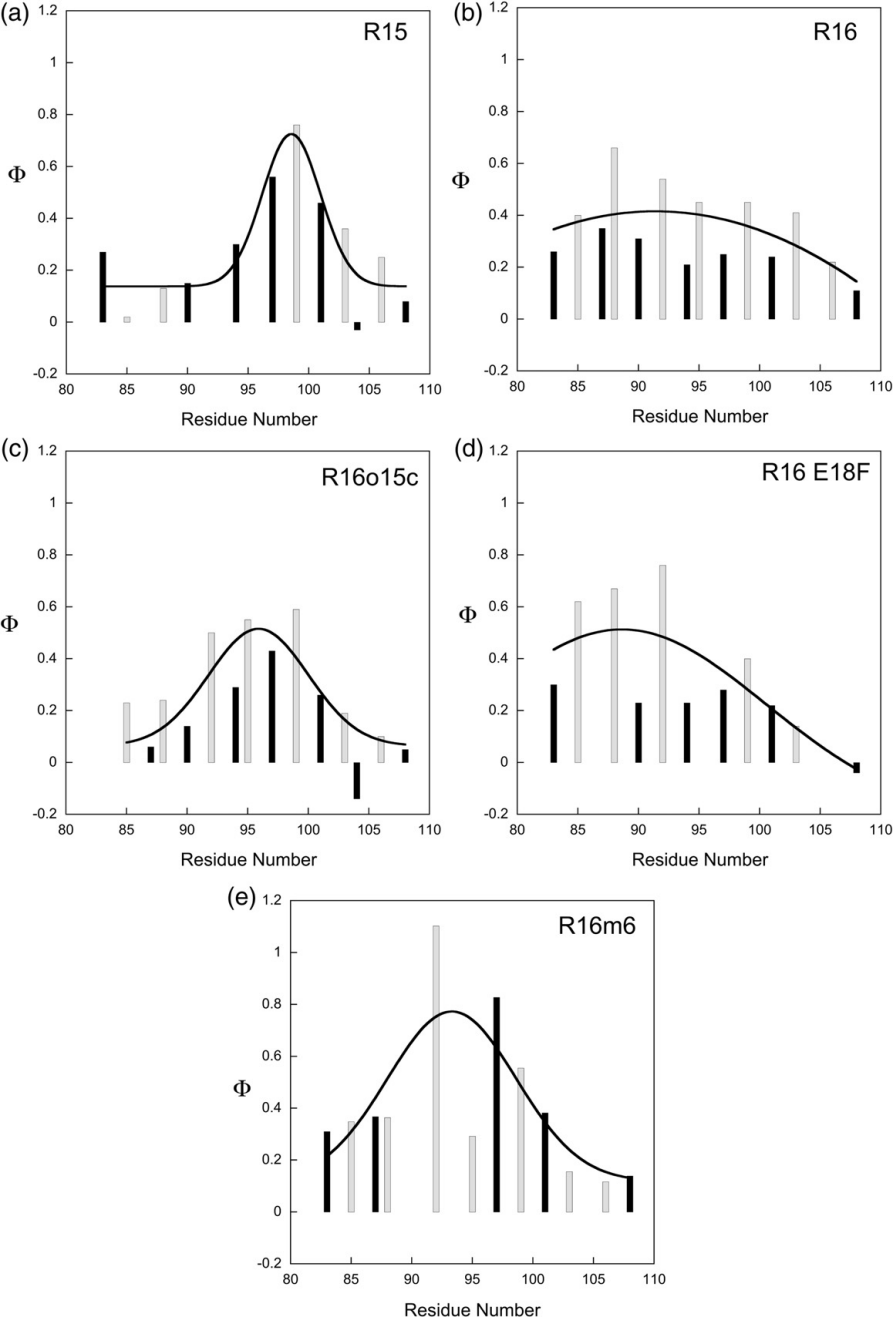
The Φ-values of R16m6(AB) are different from those of R16 but are similar to those of R15. Histograms of the TS1 Φ-values that have been determined for the C-helix of (a) R15,^7^ (b) R16,^4^ (c) R16o15c,^6^ (d) R16 E18F^14^ and (e) R16m6. Dark bars indicate core mutations probing tertiary structure, and pale bars indicate surface mutations probing helix formation. The black continuous lines show the best Gaussian fits to the data, and the corresponding best‐fit parameters are shown in Table S4. If the protein folds via a nucleation–condensation mechanism, then a range of Φ-values are expected along the helix, peaking around the nucleation site. However, if a framework mechanism is in operation, then the values will be more consistent along the helix and a Gaussian function would be a poor fit to the data. Although the C‐helix of R16m6 has an identical sequence with R16, the Φ-values are statistically similar to those of R15 (*p* = 0.007, *n* = 12). The Φ-values of R16m6(AB) are different from those of R16 but are similar to those of R15. Bar charts of the TS1 Φ-values that have been determined for the C-helix of (a) R15,^7^ (b) R16,^4^ (c) R16o15c,^6^ (d) R16 E18F^14^ and (e) R16m6. Dark bars indicate core mutations probing tertiary structure, and pale bars indicate surface mutations probing helix formation. The black continuous lines show the best Gaussian fits to the data, and the corresponding best‐fit parameters are shown in Table S4. If the protein folds via a nucleation–condensation mechanism, then a range of Φ-values are expected along the helix, peaking around the nucleation site. However, if a framework mechanism is in operation, then the values will be more consistent along the helix and a Gaussian function would be a poor fit to the data. Although the C‐helix of R16m6 has an identical sequence to that of R16, the Φ-values are statistically similar to those of R15 (*p* = 0.007, *n* = 12).

Φ-Values for TS1 were calculated as described in Materials and Methods (Fig. S4 and Table S3). There are two core mutations that give rise to nonstandard values of Φ (Φ > 1 or Φ < 0), F90A and W94F (Fig. S4b). Both show a reduction in both folding and unfolding rate constants upon mutation. They have been excluded from the following Φ-value analysis but will be discussed below. The R16m6(AB) Φ-values are shown in Fig. 5 along with the TS1 Φ-values of R15, R16, R16o15c and R16 E18F.^4,6,7,14^ Despite the C-helix of R16m6(AB) having an identical sequence with that of R16, qualitatively, the pattern of Φ-values is clearly different. Notably, there is no longer a clear distinction between the surface and core Φ-values, with the former being consistently larger, as is the case for R16 (*p* = 0.007). In fact, in R16m6(AB), the magnitude of both types of Φ-values increases substantially in the center of the helix and decreases considerably at the C-terminal end of the helix.

This comparison can be made more quantitative. The C-helix Φ-values for R15, R16o15c and R16m6(AB) can all be fitted to a single Gaussian peak with similar peak positions (around residues 99, 96 and 93, respectively) and widths (around 2, 4 and 5 residues, respectively). In addition, the peak height and basal value agree within fitting error (Table S4). In contrast, the C-helix Φ-values for R16 cannot be well fit by a Gaussian. There is a larger fitting error in the peak position (91 ± 4), and the fitting errors for the other variables are significantly larger than the estimated values themselves. The pattern of Φ-values for the R16m6 is therefore much more similar to that of R15 than R16. Indeed, the individual Φ-values for R16m6 are better correlated with R15 values (*p* = 0.039, *n* = 9) than with R16 values (*p* = 0.13, *n* = 12) despite the absolute sequence identity for the C-helix with the latter. Critically, changing just six residues, none of which are present within the C-helix itself, has changed the apparent pattern of Φ-values and, by implication, the folding mechanism. Note that stabilization of TS by the mutation E18F, which speeds both folding and unfolding significantly, does NOT change the Φ-value pattern in the manner seen in the core-swapped proteins.^14^ Instead, the Φ-values are very well correlated (pairwise) with the parent protein R16 (*p* = 0.001, *n* = 11).^14^

## Discussion

**Fig. 6:**
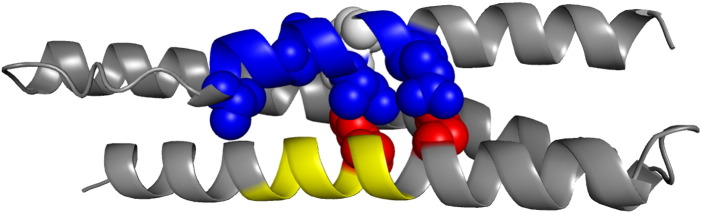
Residues important to the folding nucleus of R16m6(AB) mapped onto the R16 structure. On the A‐helix, the region of the five minimal core residues (18–29) is colored blue, with the five residues shown as blue space-filling models and Trp21 shown in white. The region of high Φ-values on the C‐helix, residues 92–99, is colored yellow, and the two nonstandard mutations, F90A and W94F, are shown as red space-filling models (see Discussion).

In R16 (and R17) secondary structure, Φ-values (probed by surface Ala–Gly mutations) are significantly higher than core Φ-values, and the Φ-values are fairly uniform along the entire length of the A‐helix and of the C‐helix.^4,5^ This pattern of Φ-values suggests a framework, diffusion–collision-like folding mechanism where partly preformed helices dock. R15 folds by a nucleation–condensation mechanism: the regions with high Φ-values in the A‐helix and in the C-helix pack together in the native structure.^7^ The minimal core residues in the A‐helix of R16m6(AB) co-localize with those residues that constitute the nucleation site on the A‐helix of R15 (shaded area in Fig. S2a). Both qualitatively and quantitatively, it can be seen that the pattern of Φ-values in R16m6(AB) is very different to its parent R16 but is remarkably similar to that of R15 (and R16o15c) (Fig. 5). In helix C of R16m6(AB), the region of high Φ-values (residues 92–99) are those that pack onto the minimal core residues in the A‐helix (Fig. 6). Given the 100% identity between the C‐helix of R16 and the C‐helix of R16m6(AB), the clear Φ-value differences in this helix are strong evidence for a change in transition‐state structure and folding mechanism in R16m6(AB). Since we know that the B-helix is essentially unfolded in the early transition state of all parent spectrin domains, we infer that the minimal core B-helix residue V65L (Φ-value of 0.1 in both R15 and R16) is not involved in the early TS in this core‐swapped protein.^4,5,7^ Thus, we propose that the minimal core residues engineered into the A‐helix of R16m6(AB) provide a folding nucleus against which the potential nucleating region in helix C can pack (Fig. 6). In this light, the nonclassical Φ-values of F90A and W94F can perhaps be understood. These large hydrophobic residues, which are at the heart of the putative nucleating region in the C‐helix (Fig. 6), slow both folding and unfolding (Fig. S4b). Perhaps, without these residues, the new nucleation site cannot be formed and these mutants are reverting to a more framework-like mechanism, associated with slower folding and unfolding.

### The relationship between frustration and folding mechanism and folding speed

Our results on the minimal core-swapped proteins described here clearly support the relationship between the shift toward a nucleation–condensation folding mechanism and the reduced landscape frustration (roughness) for these spectrin domains.^6^ R16m6(AB) behaves in a manner similar to that of R16o15c, the full R16-based core swap, including an intermediate dependence of the folding and unfolding rate constants on solvent viscosity. We have shown that the origin of these behavioral shifts in R16m6(AB) and R16o15c has been narrowed down to five mutations in the A‐helix, E18F, E19D, I22L, K25V and V29L.

Just these five key substitutions are sufficient to shift the folding mechanism of R16 toward one much more similar to R15. We propose that it is this change in mechanism that is responsible for the reduced roughness of R16m6(AB), R16o15c and R17o15c relative to R16 and R17.^6^ In the folding of R16 and R17, the long spectrin helices partially preform and must then dock as the domain crosses the transition state. We hypothesize that it is the frustrated search for the correct register (repeated cycles of misdocking and undocking) that is manifested as internal friction. Once a good nucleation site is engineered into the A‐helix of R16 or R17, this nucleus allows the correct docking of the nucleation site in helix C and, thus, sets up the correct register for A‐helix and C-helix docking, enabling more rapid folding across a less frustrated energy landscape. The internal friction slows the folding of R16 and R17 by about 5-fold.^14^ Added to this is the effect of the individual mutations E18F and K25V that speed folding significantly (by ~ 40‐fold when combined) but without reducing the internal friction, and the Φ-value analysis of E18F shows that the folding mechanism of R16 is unaltered in this fast-folding variant (Ref. 14; see Table S5).

The same five mutations are also sufficient to significantly increase the folding and unfolding rate constants of R17 (Fig. 3b) and are a subset of the residues mutated to produce R17o15c that displays faster‐folding kinetics and a smooth, R15-like folding landscape.^6^

R15, R16o15c and now R16m6(AB) all fold and unfold by a nucleation–condensation mechanism on energy landscapes that are significantly less frustrated at TS1 than is R16. Interestingly, in the three-helix‐bundle homeodomain family, studied by Fersht *et al.*, the faster‐folding members fold by a diffusion–collision mechanism, with docking of very well formed helices. Nucleation condensation is significantly slower,^22,32–36^ perhaps due to the necessity to form entropically more unfavorable long-range interactions. We suggest that the difference between the two systems is due to the length of the helices. In the homeodomains, the helices are very short (2–4 turns/helix), whereas in spectrin domains, the helices are 6–10 turns long, making the search for the correct docking register more difficult in the absence of a strong nucleation site.

There is other evidence to suggest that such misdocking events may indeed occur, from all-atom unfolding simulations performed in the Daggett laboratory.^5^ In these simulations, short-lived contiguous helical segments were seen in the denatured state and “a number of helical docking events are observed”. These docking events were only seen to occur between helices A and C, and they were “always out of register”. Importantly, these events never lead to rearrangement and correct folding, although their typical lifetime was “100s of ps”.

An important requirement of any hypothesis for the origin of the internal friction seen for R16 and R17 is that it must provide an explanation for the localization of the roughness at TS1 that has been observed along the reaction coordinate (B. G. Wensley and J. Clarke, unpublished data). Since spectrin domains fold via two consecutive transition states, we have been able to determine the roughness in both TS1 (described here), where the helices dock and topology is established, and TS2, where the protein becomes more structured.^4,5^ The denaturant dependence of the rate constants of R16 and R17 for folding and unfolding over TS2 is very strongly dependent on solvent viscosity (i.e., early internal friction is lost at TS2) (B. G. Wensley and J. Clarke, unpublished data). This is consistent with our proposal that the frustration at TS1 is due to helix misdocking as the TS is traversed and the helix register is established. Once the helices are correctly docked, folding proceeds rapidly along a smooth, unfrustrated landscape as TS2 is traversed.

While the importance of this nucleus in the A‐helix to the unfrustrated folding of these spectrin domains is clear, the nucleation mechanism is not known. Does the A‐helix nucleate first, capturing the C‐helix by the transition state, or do the two form concomitantly? Although the five key mutations are in the region of R15 with the greatest helical propensity, as determined by AGADIR, they do not significantly alter the low helix propensity shown by R16 and R17 in this region, and merely increasing the helical propensity of R16 in this region does not alter the folding kinetics significantly.^3,7^ The exact residue set involved in inducing the formation of the nucleus is unknown. An all-by-all search would take 30 variants and viscosity analyses and be prohibitively time consuming. A more productive approach must be one where simulations guide experiment, although simulations of these spectrin domains are not easy.^5^

### Conclusions: the folding landscape of R15, R16 and R17

The relative ease with which we have manipulated the folding route taken by R16 and R17 suggests that, in addition to the unusual roughness seen at TS1, the energy landscapes must be complex with at least two potential routes across them. In the absence of clear nucleating signals, the wild-type proteins access a framework-like mechanism pathway involving a frustrated search for the correct docking. However, just a few substitutions allow access to faster folding by a non-frustrated nucleation–condensation pathway, which is very similar to the one traversed by R15. In wild-type R16 and R17, this latter path is not preferred, presumably because the putative nucleation site is unstable. In producing the two full core swaps and all eight minimal core swaps, we have altered this landscape, stabilized the A-helix to C-helix folding nucleus and allowed access to an alternate pathway. The intermediate roughness observed for R16o15c and R16m6(AB) contrasts with the more complete loss of internal friction seen for R17o15c and may indicate that, in the R16-derived core swaps, the switch to the nucleation–condensation route is not complete.^22^ It is not, however, possible to determine if this is due to the use of some intermediate route or to persistent nonnative interactions that are not removed in any of our R16-based core-swapped proteins.

The idea that nonnative interactions might introduce kinetic traps in an energy landscape is not new; such kinetic traps have been observed as intermediates with some nonnative contacts that need to unfold before folding can be completed (in the immunity proteins, for example, see Ref. 37, and even in simulations using simple Gõ models^38^). However, in our case, the kinetic traps are associated with small energy barriers—the roughness we observe is not associated with accumulation of intermediates. Our results pose a further conundrum: what is the nature of the proposed rearrangement events that result in escape from the misdocked kinetic traps? Escape is apparently viscosity independent, thus unlikely to require large movements of the polypeptide chain. We hope that our experiments will stimulate simulations to investigate this question.

It is worth noting that viscosity analyses have been undertaken for several small domains with a number of folds. One of these, GCN4-p2′, has elongated helices reminiscent of the spectrin domains, but it does not show any evidence for internal friction.^20^ To date, no other domains that fold on a comparable millisecond‐to‐second timescale have been shown to have a frustrated landscape.^16–21^ However, given that this frustration may result in only relatively small (here, about 5-fold) changes in folding and unfolding rates constants,^14^ few experimental studies have been performed; thus, we cannot know how common this phenomenon may be across fold space.

## Materials and Methods

Synthetic genes for R16m7 and R17m7 were purchased from GenScript and inserted into the modified pRSETA vector used to express all of our spectrin domains. All mutagenesis, protein expression and purification methods have been described elsewhere as have details of how biophysical data are collected for these spectrin domains.^3^ The minimal core-swapped domains were treated in a manner analogous to that taken with their respective parental domains.

All equilibrium denaturation curves were fitted well to a two-state transition; fitting the kinetic data, however, was more complex.^39^ The chevron plots for R16m6(AC), R16m5, R16o15c, R17m7, R17m6(AB), R17m6(AC) and R17m5 had linear arms and, thus, were fitted to the two-state model

(2) $$\text{ln}k_{\text{obs}}=\text{ln}\left(k_{\text{f}}^{{\text{H}}_{2}\text{O}}\left(-m_{k\text{f}}\left[\text{urea}\right]\right)+k_{\text{u}}^{{\text{H}}_{2}\text{O}}\left(m_{k\text{u}}\left[\text{urea}\right]\right)\right)$$

where *k*_obs_ is the observed rate constant, *k*_f_^H_2_O^ is the folding rate constant in water, *m*_*k*f_ is the folding *m*-value, *k*_u_^H_2_O^ is the unfolding rate constant in water and *m*_*k*u_ is the unfolding *m*-value. R16m7 and R16m6(AB) displayed observable downward curvature in both chevron arms. In all cases, curvature in the refolding arm was removed by eye prior to fitting. R16m7 and R16m6(AB) could not be fitted to the sequential transition‐state model usually applied to R16 as they exhibit reduced *m*_D–N_ values.^3,40,41^ Consequently, a broad transition‐state model, which has also been successfully used to fit the R16 Φ-value data set, was employed for all chevrons collected in urea.^13,42–46^ This model incorporates a second-order polynomial into the two-state model to account for the curvature. This term, *m*′, was only added to the unfolding arm as the refolding arms were limited to the linear region only.

*k*^∆*G* = 0^, the rate constant at ∆*G*_D–N_ = 0.0 kcal mol^−^ ^1^, that is, *k*_f_ = *k*_u_, was determined using both thermodynamic and kinetic data. The [urea] at which ∆*G*_D–N_ = 0.0 kcal mol^−^ ^1^ was determined using stability measurements. Due to small deviations between kinetic and equilibrium ∆*G*_D–N_ and *m*_D–N_ values, at this concentration of urea, *k*_f_ was similar but not always identical to *k*_u_. Consequently, refolding data only (*k*_f_^H_2_O^ and *m*_*k*f_) were used to determine *k*^∆*G* = 0^. However, if unfolding data only (*k*_u_^H_2_O^ and *m*_*k*f_) or ∆*G*_D–N_ = 0.0 kcal mol^−^ ^1^ is determined kinetically, the same results, within error, are seen.

The methodology used for the viscosity analysis of R16m6(AB) was based on that previously optimized with R15, R16, R17 and R16o15c, adding solvent viscosity using 0.0 M, 0.5 M, 1.0 M and 1.5 M glucose and making chevron plots using GdmCl.^6,16–18,20,21,25,26,28^ The chevron arms collected were very short due to a combination of the limit of our stopped-flow apparatus (*k*_max_ ~ 600 s^−^ ^1^) and the use of the alternative denaturant GdmCl. Consequently, all curvature was removed from the data set and each chevron was fitted individually to a two-state model. Equilibrium and kinetic data did not agree due to inaccuracies in fitting such short chevron arms; thus, chevron plots only were used to determine *k*_f_ and *k*_u_ at ∆*G*_D–N_ = 1.5 kcal mol^−^ ^1^ and *k*_f_ = *k*_u_ at ∆*G*_D–N_ = 0.0 kcal mol^−^ ^1^. A consequence of this is that both the slope and σ-values for *k*_f_ and *k*_u_ at ∆*G*_D–N_ = 1.5 kcal mol^−^ ^1^ are identical; thus, only the *k*_f_ data have been used. Solvent viscosities at the relevant isostability, as well as the slope of the viscosity plots and magnitude of σ, were measured and calculated as previously described.^6^

Briefly, since, from Eq. (1), at isostability (i.e., constant ∆*G*^TS^), $k\propto \frac{C}{\eta +\sigma }$, a plot of 1/*k versus* solvent viscosity (η) allows internal friction (σ) in centipoises to be determined and the effect of σ on *k* to be evaluated.

The R16m6(AB) Φ-value chevrons were globally fitted, and the wild-type value quoted in Table 1 and Table S1 comes from this global fit. In the global fit, *m*_*k*f_ and *m*′ were shared. The equilibrium free energy in water, *ΔG*_D − N_^H_2_O^, was calculated for each mutant from the equilibrium data using

(3) $$\Delta G_{\text{D}-\text{N}}^{{\text{H}}_{2}\text{O}}={\left[\text{urea}\right]}_{50\%}\langle m_{\text{D}-\text{N}}\rangle$$

where [urea]_50%_ is the [urea] where [D] = [N] and 〈*m*_D–N_〉 is the mean *m*_D–N_ of 39 mutants and was 1.49 kcal mol^−^ ^1^ M^−^ ^1^. From these, *ΔΔG*_D − N_^H_2_O^ was calculated for each Φ-value pair. Φ-Values were calculated from folding data using

[4] $$\Phi =\frac{\Delta \Delta G_{\text{D}-\text{TS}}}{\Delta \Delta G_{\text{D}-\text{N}}}=\frac{RT\ln\left(\frac{k_{{}_{\text{f}}\text{,WT}}^{{\text{H}}_{2}\text{O}}}{k_{{}_{\text{f}}\text{,mut}}^{{\text{H}}_{2}\text{O}}}\right)}{\Delta \Delta G_{\text{D}-\text{N}}}$$

where *k*__f_,WT_^H_2_O^ and *k*__f_,mut_^H_2_O^ are the folding rate constants in water for the wild type and mutant, respectively.^48^ Φ was only calculated where *ΔΔG*_D − N_^H_2_O^ ≥ 0.5 kcal mol^−^ ^1^. As *m*_*k*f_ was shared, the value of Φ is invariant with [urea].

## Figures and Tables

**Table 1:** Selected parameters for the R16 and R17 minimal core variants

Domain	*m*_D − N_^eqb^ (kcal mol^−^ ^1^ M^−^ ^1^)	Δ*G*_D − N_^H_2_O^ (kcal mol^−^ ^1^)	*k*^Δ*G* = 0^ (s^−^ ^1^)
R15^a^	1.8 (± 0.1)	6.8 (± 0.2)	50 (± 20)
R16^a^	1.9 (± 0.1)	6.3 (± 0.2)	0.19 (± 0.01)
R17^a^	2.0 (± 0.1)	6.1 (± 0.2)	3.0 × 10^−^ ^2^ (± 3 × 10^−^ ^3^)
R16o15c^a^	1.9 (± 0.1)	5.5 (± 0.2)	12 (± 2)
R17o15c^a^	1.7 (± 0.1)	3.3 (± 0.2)	10 (± 1)
R16m7	1.5 (± 0.1)	6.2 (± 0.1)	14 (± 4)
R16m6(AC)	1.7 (± 0.1)	5.7 (± 0.1)	28 (± 9)
R16m6(AB)	1.5 (± 0.1)	5.9 (± 0.1)	20.9 (± 0.8)
R16m5	1.5 (± 0.1)	4.6 (± 0.1)	50 (± 10)
R17m7	1.5 (± 0.1)	3.9 (± 0.1)	13 (± 4)
R17m6(AC)	1.4 (± 0.1)	3.1 (± 0.1)	20 (± 10)
R17m6(AB)	1.4 (± 0.1)	3.6 (± 0.1)	21 (± 5)
R17m5	1.4 (± 0.1)	2.9 (± 0.1)	22 (± 5)

See Table S1 for other thermodynamic and kinetic parameters and Materials and Methods for fitting details. See Table S1 for other thermodynamic and kinetic parameters and Materials and Methods for fitting details.

a R15, R16 and R17 data are taken from Ref. 3, and the R16o15c and R17o15c data are taken from Ref. 6 and are included for comparison.
