# Supplementary material for: Protein Folding: Adding a Nucleus to Guide Helix Docking Reduces Landscape Roughness
Source: J Mol Biol. 2012 Oct 26;423(3):273–83. doi: 10.1016/j.jmb.2012.08.003 (PMC3469821; doi:10.1016/j.jmb.2012.08.003)
Supplement: Supplementary file 1 — Supplementary data [file mmc1.pdf]

Supplementary Information for:

Protein folding: Adding a nucleus to guide helix docking reduces landscape roughness

Beth G. Wensley, Lee Gyan Kwa, Sarah L. Shammas, Joseph M. Rogers and Jane Clarke.

This Supplementary File contains Supplementary Tables 1-5, Supplementary Figures 1-4 and associated References

Table S1 Full equilibrium and kinetic parameters for the R16 and R17 minimal core variants

Domain	$[Den]_{50\%}$ (M)	m^{eqb}_{D-N} (kcal mol^{-1} M^{-1})	$\Delta G^{H_2O}_{D-N}$ (kcal mol^{-1})	$k^{H_2O}_{f}$ (s^{-1})	m_{k_f} (M^{-1})	$k^{H_2O}_{u}$ (s^{-1})	m_{k_u} (M^{-1})	$\Delta G^{H_2O}_{kin}$ (kcal mol^{-1})	$k^{\Delta G=0}$ (s^{-1})
R15[a]	3.8 (±0.1)	1.8 (±0.1)	6.8 (±0.2)	6.0×10^4 ($\pm 1.3 \times 10^4$)	1.8 (±0.1)	1.31 (±0.22)	1.0 (±0.1)	6.3 (±0.2)	50 (±20)
R16[a]	3.3 (±0.1)	1.9 (±0.1)	6.3 (±0.2)	126 (±2)	2.0 (±0.1)	3.2×10^{-3} ($\pm 1 \times 10^{-4}$)	1.2[b]	6.3 (±0.3)	0.19 (±0.01)
R17[a]	3.1 (±0.1)	2.0 (±0.1)	6.1 (±0.2)	30 (±2)	2.3 (±0.1)	4.0×10^{-4} ($\pm 3 \times 10^{-5}$)	1.5 (±0.1)	6.6 (±0.3)	3.0×10^{-2} ($\pm 3 \times 10^{-3}$)
R16o15c[a]	2.9 (±0.1)	1.9 (±0.1)	5.5 (±0.2)	2200 (±300)	1.8 (±0.1)	0.56 (±0.06)	1.1 (±0.1)	4.9 (±0.1)	12 (±2)
R17o15c[a]	1.8 (±0.1)	1.7 (±0.1)	3.3 (±0.2)	141 (±5)	1.3 (±0.1)	0.75 (±0.04)	1.7 (±0.1)	3.1 (±0.1)	10 (±1)
R16m7[c]	4.2 (±0.1)	1.5 (±0.1)	6.2 (±0.1)	7100 (±1100)	1.2 (±0.1)	0.04 (±0.01)	1.6 (±0.1)	7.2 (±0.2)	14 (±4)
R16m6(AC)	3.4 (±0.1)	1.7 (±0.1)	5.7 (±0.1)	2600 (±420)	1.3 (±0.1)	1.6 (±0.3)	0.8 (±0.1)	4.4 (±0.2)	28 (±9)
R16m6(AB)[d]	3.9 (±0.1)	1.5 (±0.1)	5.9 (±0.1)	1.37×10^4 ($\pm 1 \times 10^2$)	1.58 (±0.02)	0.15 (±0.01)	1.50 (±0.01)	6.77 (±0.01)	20.9 (±0.8)
R16m5	3.0 (±0.1)	1.5 (±0.1)	4.6 (±0.1)	4300 (±700)	1.5 (±0.1)	6.83 (±0.09)	0.7 (±0.1)	3.8 (±0.1)	50 (±10)
R17m7	2.7 (±0.1)	1.5 (±0.1)	3.9 (±0.1)	460 (±72)	1.3 (±0.1)	2.3 (±0.4)	0.8 (±0.1)	3.1 (±0.1)	13 (±4)
R17m6(AC)	2.3 (±0.1)	1.4 (±0.1)	3.1 (±0.1)	100 (±20)	0.7 (±0.2)	5.5 (±2.7)	0.8 (±0.1)	1.7 (±0.3)	20 (±10)
R17m6(AB)	2.6 (±0.1)	1.4 (±0.1)	3.6 (±0.1)	210 (±20)	0.9 (±0.1)	5.1 (±1.1)	0.7 (±0.1)	2.2 (±0.1)	21 (±5)
R17m5	2.1 (±0.1)	1.4 (±0.1)	2.9 (±0.1)	120 (±10)	0.8 (±0.1)	6.2 (±1.4)	0.9 (±0.1)	1.8 (±0.1)	22 (±5)

[a] R15, R16, R17, R16o15c and the R17o15c data are included for comparison and are taken from [1; 2]. The R17o15c kinetics were globally fitted to a broad transition state model, where the quadratic term was -6.3×10^{-2} ($\pm 7.0 \times 10^{-3}$) M^{-1}.

[b] This is the globally fitted R16 unfolding *m*-value determined from the R16 Φ-value data set [3].

[c] The R16m7 kinetics were fitted to a broad transition state model, where the quadratic term was -6×10^{-2} ($\pm 1 \times 10^{-2}$) M^{-1}.

[d] The R16m6(AB) kinetic data shown are the wild-type data from the global fit used in the Φ-value analysis (see Materials and Methods for details).

Table S2 Viscosity parameters[a]

Domain	**Slope**	error	**σ (cP)**	error
R15	**0.75**	0.03	**0.26**	0.09
R16	**0.20**	0.03	**3.9**	0.8
R17	**0.15**	0.03	**6.4**	2.7
R16o15c	**0.38**	0.02	**2.1**	0.2
R16m6(AB)[b]	**0.39**	0.02	**1.6**	0.2

[a] Analysis as described and all data except for R16m6(AB) taken from [2]. Weighted averages of the fitted slopes and σ values were calculated using k at $\Delta G_{\text{D-N}}$ = 0.0 kcal mol^{-1} and k_f and k_u at $\Delta G_{\text{D-N}}$ = 1.5 kcal mol^{-1}

[b] Kinetic data only were used in the analysis of R16m6(AB). The slope and σ parameters at $\Delta G_{\text{D-N}}$ = 1.5 kcal mol^{-1} are identical when calculated using either k_{f} or k_{u} data, so only one is used in calculating the weighted average.

Table S3 Thermodynamic and kinetic parameters for Φ-value R16m6(AB) mutants

Mutant	$[\text{urea}]_{50\%}$ (M)	$m_{\text{D-N}}$ (kcal mol^{-1} M^{-1})	$\Delta G^{\text{H}_2\text{O}}_{\text{D-N}}$ (kcal mol^{-1})[a]	$\Delta\Delta G^{\text{H}_2\text{O}}_{\text{D-N}}$ (kcal mol^{-1})[b]	$k_f^{\text{H}_2\text{O}}$ (s^{-1})	Φ_f [b,c,d]
WT	3.93 (±0.1)	1.35 (±0.03)	5.9 (±0.1)	-	13730 (±80)	-
Core Mutants						
I83A	3.13 (±0.1)	1.52 (±0.05)	4.7 (±0.1)	1.2 (±0.1)	6900 (±400)	0.34
L87A	2.37 (±0.1)	1.59 (±0.02)	3.5 (±0.1)	2.3 (±0.1)	3100 (±100)	0.38
F90A	3.89 (±0.1)	1.66 (±0.03)	5.8 (±0.1)	0.1 (±0.1)	7740 (±70)	-
W94F	4.26 (±0.1)	1.65 (±0.02)	6.3 (±0.1)	-0.5 (±0.1)	8750 (±80)	-0.55[e]
L97A	3.34 (±0.1)	1.63 (±0.03)	5.0 (±0.1)	0.9 (±0.1)	3970 (±40)	0.83
A101G	2.85 (±0.1)	1.59 (±0.02)	4.2 (±0.1)	1.6 (±0.1)	4610 (±30)	0.40
R104A	4.06 (±0.1)	1.50 (±0.03)	6.0 (±0.1)	-0.2 (±0.1)	14910 (±70)	-
L108A	3.62 (±0.1)	1.39 (±0.02)	5.4 (±0.1)	0.5 (±0.1)	12510 (±80)	0.12
Surface Mutants						
Q85A	4.07 (±0.1)	1.39 (±0.02)	6.1 (±0.1)	-	16100 (±100)	-
Q85G	3.75 (±0.1)	1.62 (±0.02)	5.6 (±0.1)	0.8 (±0.1)	10290 (±60	0.34
A88G	3.49 (±0.1)	1.55 (±0.02)	5.2 (±0.1)	1.2 (±0.1)	6850 (±40)	0.36
D92A	4.37 (±0.1)	1.46 (±0.02)	6.5 (±0.1)	-	35200 (±200)	-
D92G	3.90 (±0.1)	1.57 (±0.02)	5.8 (±0.1)	0.6 (±0.1)	12750 (±90)	1.09
K95A	3.85 (±0.1)	1.53 (±0.02)	5.7 (±0.1)	-	10268 (±60)	-
K95G	3.42 (±0.1)	1.58 (±0.02)	5.1 (±0.1)	1.3 (±0.1)	5370 (±30)	0.30
Q99A	4.24 (±0.1)	1.60 (±0.02)	6.3 (±0.1)	-	21800 (±200)	-
Q99G	3.69 (±0.1)	1.66 (±0.02)	5.5 (±0.1)	0.9 (±0.1)	9990 (±60)	0.53
A103G	3.74 (±0.1)	1.55 (±0.02)	5.6 (±0.1)	0.8 (±0.1)	11250 (±70)	0.15
Q106A	4.10 (±0.1)	1.56 (±0.02)	6.1 (±0.1)	-	15800 (±90)	-
Q106G	3.73 (±0.1)	1.52 (±0.02)	5.6 (±0.1)	0.8 (±0.1)	13670 (±80)	0.11

[a] The value given is taken from equilibrium denaturation experiments. $\Delta G^{\text{H}_2\text{O}}_{\text{D-N}}$ is calculated from $\Delta G^{\text{H}_2\text{O}}_{\text{D-N}} = m_{\text{D-N}}[\text{urea}]_{50\%}$, using a mean $m_{\text{D-N}}$ value of 1.49 kcal mol^{-1}.

[b] For Ala-Gly helix scanning positions values of $\Delta\Delta G^{\text{H}_2\text{O}}_{\text{D-N}}$ and Φ are shown against the appropriate glycine mutant.

[c] Φ-values are only calculated for mutants where $\Delta\Delta G^{\text{H}_2\text{O}}_{\text{D-N}} \geq 0.5$ kcal mol^{-1} [4].

[d] Errors in the Φ-values were propagated from errors of the fits of the chevron plots and the $\Delta\Delta G^{\text{H}_2\text{O}}_{\text{D-N}}$ values and are ≤ 0.1.

[e] This Φ-value pair show non-standard behavior and has been excluded from the Φ-value analysis. See Discussion. (F90A also shows non-standard behavior, but it has $\Delta\Delta G^{\text{H}_2\text{O}}_{\text{D-N}} < 0.5$ kcal mol^{-1}.)

Table S4. Fitting of the Φ-values to a Gaussian curve (See Figure 3 in main text) shows that R16 core-swap mutants R16o15c and R16m6(AB) Φ-value patterns are similar to R15 and very different to R16.

	Peak position (p)			Peak width (w)			Peak height (h)			Basal Φ-value (Φ_0)		
R15	98.6	±	0.5	3.4	±	0.8	0.59	±	0.11	0.14	±	0.05
R16	91.4	±	3.8	200	±	59300	40	±	25000	-40	±	25000
R16o15c	95.9	±	1.0	5.8	±	2.2	0.45	±	0.11	0.06	±	0.10
R16 E18F	88.6	±	4.0	17	±	20	0.7	±	0.9	-0.2	±	0.9
R16m6	93.3	±	1.2	7.5	±	3.4	0.66	±	0.22	0.12	±	0.21

R15, which has a pattern of Φ-values that are typical of nucleation condensation (with high Φ-values at a nucleation point decreasing away from that nucleus), can be fit to a Gaussian (see equation 1 below) distribution of Φ-values with the nucleus, in the C-helix, centered around residues 98-99. In R16 and R16 E18F, no clear nucleation pattern of Φ-values is observed. In the core-swap varieties of R16 the distribution of Φ-values is more similar to that of R15, even though the sequence of the C-helix in R16m6(AB) is identical to that of R16.

Φ-values from each of the domains were fit to equation 1 below, where R is the residue number:

$$\Phi = \Phi_0 + h\exp^{\frac{-(\mathrm{R}-p)^2}{w^2}} \qquad [1]$$

Table S5 Single mutations can speed the folding of R16 but do not increase the internal friction.

Comparison of the folding rate constants and the internal friction of wild-type R15 and R16, and the single point mutants R16 E18F and K25V.

Domain	$\Delta G_{D-N}^{H_2O}$ (kcal mol^{-1})	k_f s	Slope of relative viscosity dependence	σ (cP)
R15[a]	6.8 ± 0.2	60000 ±13000	0.75 ± 0.03	0.26 ± 0.09
R16[a]	6.4 (±0.2)	126 (±2)	0.20 ± 0.03	3.9 ± 0.8
R16 E18F	6.5 (±0.1)	2200 (±200)	0.23 ± 0.03	3.8 ± 1.1
R16 K25V	6.4 (±0.1)	630 (±50)	0.15 ± 0.03	6.3 ± 1.5

R15 and R16 data taken from [2] Mutant data taken from [5].

Slopes are calculated from fitting all two/three datasets together

σ reported is the weighted average from the two/three individual estimates

Supplementary Figures

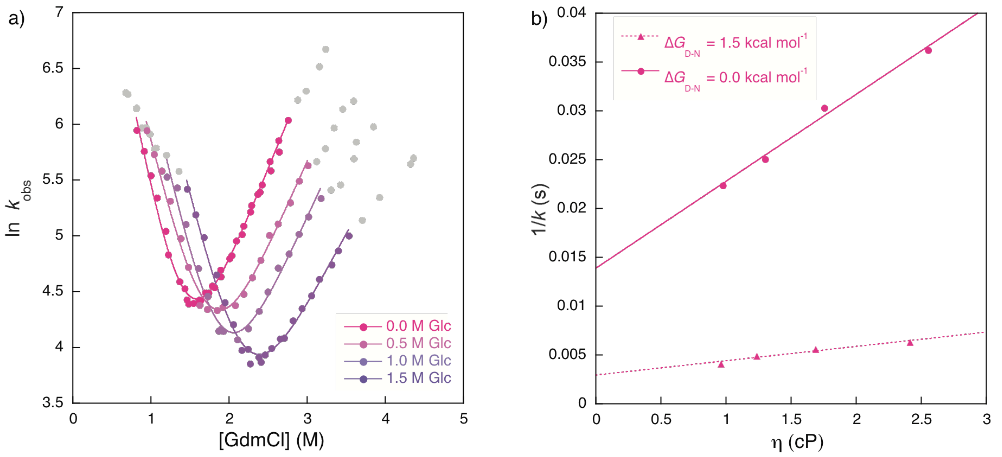

Figure S1 The effect of increasing concentrations of glucose (Glc) on the folding and unfolding of R16m6(AB)

(a) Chevron plots and fits at varying Glc concentrations. All observed curvature was excluded from fitting and is shown as grey circles. (b) σ plot for R16m6(AB). See the Supplementary Materials in Wensley *et al.* [2] for details of how to extract σ from these data .

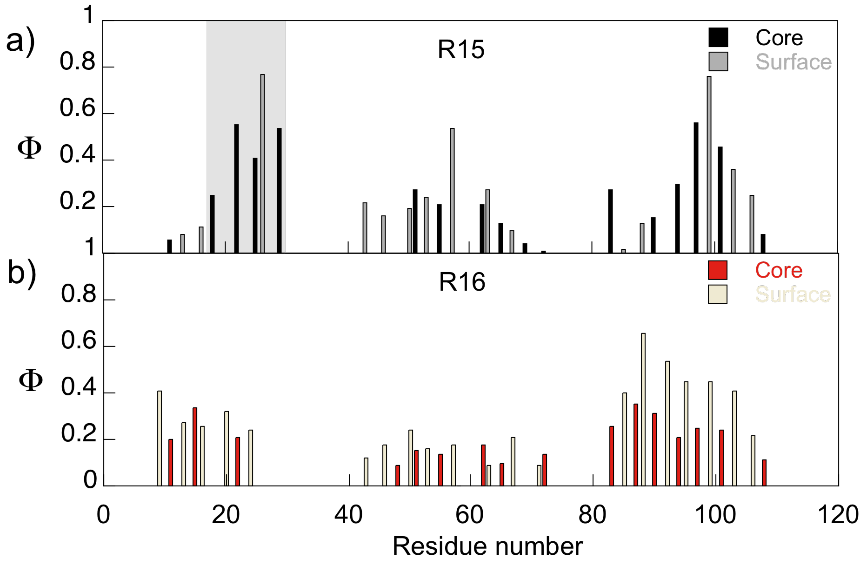

Figure S2 Comparison of the Φ-values of R15 and R16

R15 data taken from [6] and R16 data from [3]. R15 has an archetypal nucleation condensation pattern of Φ-values with high Φ-values at the nucleus and with secondary (surface) and tertiary (core) Φ-values showing the same pattern. The high Φ-values in the A-helix and the C-helix are in parts of the helices that pack against each other, to form the folding nucleus around which structure condenses. The Φ-value pattern of R16, on the other-hand suggests a folding mechanism closer to diffusion collision. The surface Φ-values are higher than the core Φ-values, suggesting that formation of secondary structure precedes that of tertiary structure. Furthermore there is no discernable nucleation region in R16. The shaded region in A shows the position of the residues in the A-helix that are changed in the minimal core-swapped versions of R16 and R17 described in this manuscript.

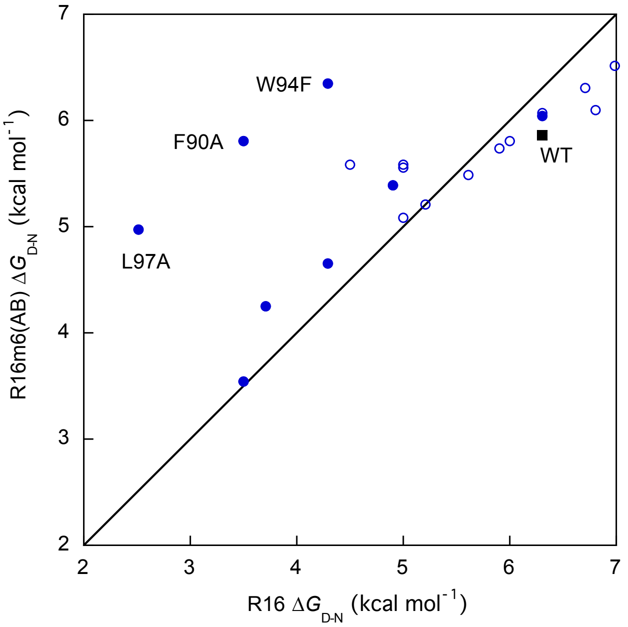

Figure S3 Mutations have the same effect in R16m6(AB) as in R16.

Free energy of wild type and variant proteins compared. Positions where the same mutation was made in both R16m6(AB) and R16 are compared. Core mutations are shown as filled circles and surface (Ala to Gly) mutations as open circles. R16 data taken from Scott *et al.* [3]. Excluding the outliers F90A, W94F and L97A a correlation between the two data sets can be seen, although perhaps a slightly smaller loss of stability is seen upon mutation in R16m6(AB) for the core mutations. The three outliers pack against the minimal core residues in the A-helix. Interestingly, the reduced in $m_{D\text{-}N}$ in R16m6(AB) relative to R16 results in a systematic increase in $[urea]_{50\%}$ in the R16m6(AB) data set.

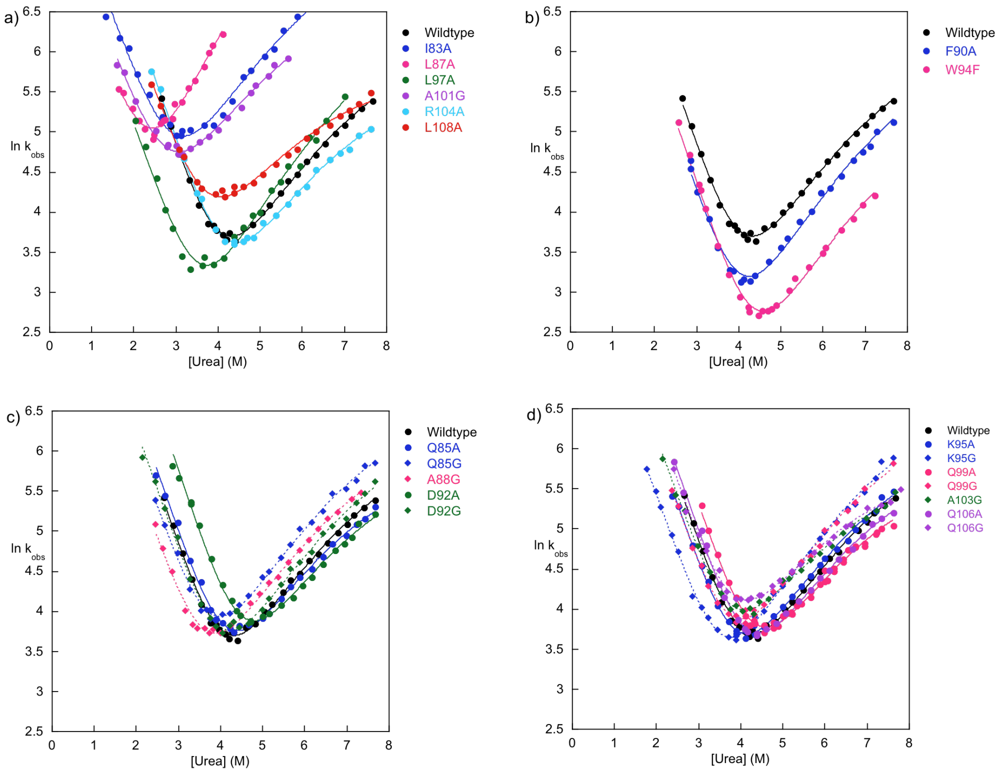

Figure S4 R16m6(AB) Φ-value analysis chevron plots and fits

(a) and (b) are the core mutations made. These were usually made to Ala, but W94A was very destabilizing and the less destabilizing W94F was substituted. (b) Shows the two mutants (F90A and W94F) that exhibited kinetics that were clearly non-standard with small changes in stability but significant reductions in both the folding and unfolding kinetics. (c) and (d) show surface mutations, where each residue was mutated to both Ala and Gly and the two compared. Any rollover seen in the refolding arm was excluded and these data points are not shown. Data points with $k > 600\ s^{-1}$ were also excluded. See Materials and Methods for details of how these data were fitted.

Supplementary References

1. Scott, K. A., Batey, S., Hooton, K. A. & Clarke, J. (2004). The folding of spectrin domains I: wild-type domains have the same stability but very different kinetic properties. *J. Mol. Biol.* **344**, 195-205.
2. Wensley, B. G., Batey, S., Bone, F. A. C., Chan, Z. M., Tumelty, N. R., Steward, A., Kwa, L. G., Borgia, A. & Clarke, J. (2010). Experimental evidence for a frustrated energy landscape in a three-helix-bundle protein family. *Nature* **463**, 685-688.
3. Scott, K. A., Randles, L. G. & Clarke, J. (2004). The folding of spectrin domains II: phi-value analysis of R16. *J. Mol. Biol.* **344**, 207-221.
4. Fersht, A. R. & Sato, S. (2004). Φ-value analysis and the nature of protein-folding transition states. *Proc. Natl Acad. Sci. USA* **101**, 7976-7981.
5. Wensley, B. G., Kwa, L., Shammas, S. L., Rogers, J. M., Browning, S., Yang, Z. & Clarke, J. (2012). Rationalizing the slow, frustrated folding of spectrin domains: separating the effects of internal friction and transition state energy. *Submitted*.
6. Wensley, B. G., Gärtner, M., Choo, W., Batey, S. & Clarke, J. (2009). Different members of a simple three-helix bundle protein family have very different folding rate constants and fold by different mechanisms. *J. Mol. Biol.* **390**, 1074-1085.
